# Easy and fast method for expression and native extraction of *Plasmodium vivax* Duffy binding protein fragments

**DOI:** 10.1186/s12936-018-2216-6

**Published:** 2018-02-08

**Authors:** Darwin Andrés Moreno-Pérez, Luis Alfredo Baquero, Maritza Bermúdez, Laura Alejandra Gómez-Muñoz, Yahson Varela, Manuel Alfonso Patarroyo

**Affiliations:** 1 Molecular Biology and Immunology Department, Fundación Instituto de Inmunología de Colombia (FIDIC), Carrera 50 No. 26–20, Bogotá, DC Colombia; 2 Livestock Sciences Faculty, Universidad de Ciencias Aplicadas y Ambientales (U.D.C.A), Calle 222 No. 55–37, Bogotá, DC Colombia; 3 Receptor-Ligand Department, Fundación Instituto de Inmunología de Colombia (FIDIC), Carrera 50, No. 26–20, Bogotá, Colombia; 4 Chemical Synthesis Department, Fundación Instituto de Inmunología de Colombia (FIDIC), Carrera 50 No. 26–20, Bogotá, DC Colombia; 5 Basic Sciences Department, School of Medicine and Health Sciences, Universidad del Rosario, Carrera 24 No. 63C-69, Bogotá, DC Colombia

**Keywords:** *Plasmodium vivax*, Reticulocyte, Duffy binding protein, Soluble extraction, Protein–cell interaction

## Abstract

**Background:**

The *Plasmodium vivax* Duffy binding protein (*Pv*DBP) has been the most studied ligand binding human reticulocytes to date. This molecule has a cysteine-rich domain in region II (RII) which has been used as control for evaluating the target cell binding activity of several parasite molecules. However, obtaining r*Pv*DBP-RII in a soluble form using the *Escherichia coli* expression system usually requires laborious and time-consuming steps for recovering the molecule’s structure and function, considering it is extracted from inclusion bodies. The present study describes an easy and fast method for expressing and obtaining several *Pv*DBP fragments which should prove ideal for use in protein–cell interaction assays.

**Results:**

Two *Pv*DBP encoding regions (*rii* and *riii/v*) were cloned in pEXP5-CT vector and expressed in *E. coli* and extracted from the soluble fraction (r*Pv*DBP-RII_S_ and r*Pv*DBP-RIII/V_S_) using a simple freezing/thawing protocol. After the purification, dichroism analysis enabled verifying high r*Pv*DBP-RII_S_ and r*Pv*DBP-RIII/V_S_ secondary structure α-helix content, which was lowered when molecules were extracted from inclusion bodies (r*Pv*DBP-RII_IB_ and r*Pv*DBP-RIII/V_IB_) using a denaturing step. Interestingly, r*Pv*DBP-RII_S_, but not r*Pv*DBP-RII_IB_, bound to human reticulocytes, while r*Pv*DBP-RIII/V_S_ and r*Pv*DBP-RIII/V_IB_ bound to such cells in a similar way to negative control (cells incubated without recombinant proteins).

**Conclusions:**

This research has shown for the first time how r*Pv*DBP-RII can be expressed and obtained in soluble form using the *E. coli* system and avoiding the denaturation and refolding steps commonly used. The results highlight the usefulness of the r*Pv*DBP-RIII/V_S_ fragment as a non-binding control for protein-cell target interaction assays. The soluble extraction protocol described is a good alternative to obtain fully functional *P. vivax* proteins in a fast and easy way, which will surely prove useful to laboratories working in studying this parasite’s biology.

## Background

*Plasmodium* merozoite invasion of human erythrocytes is a complex process requiring the interaction of specific molecules, i.e. parasite ligands and cell receptors [1]. Only one route used by *Plasmodium vivax* for invading reticulocytes, in which the Duffy binding protein (*Pv*DBP) participates, has been experimentally confirmed to date; such molecule is located in the parasite’s micronemes and interacts with the Duffy antigen receptor for chemokines (DARC) located on erythrocyte membrane [2, 3]. *Pv*DBP belongs to a family of erythrocyte binding proteins. These have two conserved regions [domains rich in cysteine (Cys) amino acids] having a high level of homology between *Plasmodium falciparum*, *P. vivax* and *Plasmodium knowlesi* [4], which would suggest a common evolutionary origin and the conservation of erythrocyte binding domains in evolutionarily distant parasite species.

Eukaryote systems have been used to produce several *Pv*DBP fragments in a native form and characterize their cell-binding activity. As an example, Chitnis et al. [5] built several vectors containing DNA sequences encoding for several *Pv*DBP regions (RI, RII, RIII/V and RVI), which were transfected in COS-7 cells with a confluence of 30–50%. The cells were cultured in a humified CO_2_ (5%) incubator at 37 °C and then were used for erythrocyte binding assays. The results showed that Duffy positive human erythrocytes (Duffy+), but not those treated with chymotrypsin (which removes DARC), formed rosettes on COS-7 cell surface expressing *Pv*DBP-RII recombinantly (r) (r*Pv*DBP-RII); this enabled identifying this domain’s role in binding. In 1999, Ranjan et al. [6] used the rosette formation assay for mapping the region within the RII domain responsible for binding to human and primate erythrocytes. The authors expressed r*Pv*DBP-RII in several fragments (including some Cys residues) on mammal COS cell surface and found that human erythrocytes were capable of forming rosettes only with cells expressing the fragment from Cys 4 to Cys 7 (*Pv*DBP region II). On the other hand, Dutta et al. [7] produced r*Pv*DBP-RII in *Spodoptera frugiperda* 21 (*Sf*21) insect cells’ culture supernatant using a bioreactor and maintaining the dissolved oxygen level at 60% of air saturation. The molecule so purified using a two-step purification protocol by FPLC was able to bind Duffy+ human erythrocytes and also induced a natural immune response blocking r*Pv*DBP-RII interaction with cells.

An *Escherichia coli* prokaryotic expression system has also been used for obtaining recombinant proteins as it is extremely cheap, easy to use and molecules can be expressed in abundance and in a short time (maximum 1 day), compared to the eukaryote system (from 3 to 4 days). Fraser et al. [8] expressed the r*Pv*DBP-RII/IV-GST fusion protein (which includes a hydrophilic Cys-free region contiguous to RII) in *E. coli* and extracted the molecule by sonication in phosphate buffer saline (PBS) (three times for 1.5–2 min each one) and purified it by the standard GST fusion method. Even r*Pv*DBP-RII/IV was obtained in soluble form and was antigenic during natural infection, the molecule had no erythrocyte binding activity. Later, Dutta et al. [7] reported that r*Pv*DBP-RII was predominantly found in the insoluble fraction (inclusion bodies (IB)) of *E. coli* culture. However, the extracted molecule was unable to bind to erythrocytes when using a denaturing step.

The only protocol for obtaining fully functional r*Pv*DBP-RII from a prokaryotic system has been described by Singh et al. The authors used the *E. coli* system for expressing r*Pv*DBP-RII and extracting it from IB using urea as denaturing agent. r*Pv*DBP-RII was subjected to a refolding step in redox conditions (called rapid dilution method) for 36 h at 10 °C, dialyzed for 48 h against PBS and purified by ion exchange chromatography and gel filtration [9]. Two milligrams per liter of culture were produced with 98% of r*Pv*DBP-RII protein purity. Biochemical and functional characterization corroborated that the molecule was immunogenic in rabbits and had suitable structural formation as r*Pv*DBP-RII specifically bound to Duffy+ erythrocytes.

Bhardwaj et al. [10], recently used an exponential feeding strategy for optimizing r*Pv*DBP-RII expression. The method was focused on using the *pvdbp*-*rii* synthetic gene with codon optimization for improving r*Pv*DBP-RII production, also taking advantage of high cell density achieved by fed-batch fermentation where the pH, stirring speed, dissolved oxygen and air flow variables were controlled. More than 95% of the protein was extracted from IB, refolded by rapid dilution method in redox conditions, as previously described, and purified by ion exchange chromatography [10]. r*Pv*DBP-RII was capable of binding to Duffy+ erythrocytes but not to cells which had been previously treated with chymotrypsin. Antibodies produced in mice managed to inhibit *Pv*DBP variants (*Pv*O, *Pv*AH and *Pv*P) binding to the DARC receptor with the same efficiency, thereby validating its functional activity.

As described, r*Pv*DBP-RII has been expressed and obtained by using eukaryote and prokaryote expression systems. However, the technology required for replicating the methods described above and producing a sufficient amount of r*Pv*DBP-RII is extremely expensive and/or complex and require several lengthy and labor-intensive steps. The present work was thus aimed at expressing and obtaining two *Pv*DBP recombinant regions (RII and RIII/V) using the pEXP5-CT vector and the *E. coli* prokaryote expression system, in soluble form, completely functional and avoiding the commonly used denaturation and refolding steps.

## Methods

### Designing primers and amplifying *dbp* gene regions by PCR

Two sets of specific primers for amplifying *P. vivax Pv*DBP protein RII (forward: 5′ ATGTCGAATGGTGGCAATCCT 3′; reverse: 5′ GGTGGCCTGAGATTTAGC 3′) and RIII/V (forward: 5′ ATGGCTAAAAATGTTGATCCGCA 3′; reverse 5′ GTTAGTTGTATCATTAGTAGTT 3′) fragments were manually designed (using Gene runner software, version 3.05) on a *pvdbp* gene sequence from the Salvador-I (Sal-I) reference strain reported in the PlasmoDB database (Gene ID: PVX_110810) [11, 12]. The gDNA (50 ng) from *P. vivax* Colombia Guaviare 1 (VCG-I) strain parasites (propagated as described previously [13]) was then used as template for 25 µL PCR reactions containing 1× enzyme, 2× KAPA HiFi HotStart ReadyMix (KAPA Biosystems, Woburn, MA, USA), 0.3 µM of primers and ultrapure water until reaction volume was completed. PCR for amplifying *pvdbp* fragments began with a denaturing step at 95 °C for 5 min, followed by 35 cycles at 98 °C for 20 s, 56 °C for 15 s and 72 °C for 1 min and a final extension step at 72 °C for 5 min. PCR products were purified using a Wizard PCR Clean-Up System kit (Promega, Madison, USA), ligated into pEXP5-CT/TOPO expression vector (Invitrogen, Carlsbad, USA) and then transformed in TOP-10 *E. coli* cells (Invitrogen).

Several recombinant clones were grown for 16 h at 37 °C at 270 rpm in Luria Bertani (LB) medium, supplemented with ampicillin (100 µg/mL), using a Lab-line Incubator Shaker. This was followed by plasmid DNA extraction using an UltraClean mini plasmid prep purification kit (MO BIO Laboratories, California, USA). After confirming the integrity of the clones obtained from the primate-adapted VCG-I strain using a ABI-3730 XL sequencer (Macrogen, Seoul, South Korea), the consensus sequence was compared to the sequence reported for the Sal-I strain *dbp* gene using ClustalW NPS software [14].

### *Pv*DBP fragment expression

Recombinant plasmids pEXP5-*pvdbp*-*rii* and pEXP5-*pvdbp*-*riii/v* were transformed in *E. coli* BL21-AI cells (Invitrogen), following the manufacturer’s recommendations. The cells were grown for 16 h at 37 °C, at 270 rpm, in 50 mL LB medium containing 100 µg/mL ampicillin. The next day, 1 L LB medium containing ampicillin was added to the initial culture, left in 1:20 dilution and then incubated in the same growth conditions as those mentioned above. Once ~ 0.8 OD_600_ had been reached, the cultures were incubated on ice for 30 min and 0.2% (w/v) l-arabinose (Sigma-Aldrich, St. Louis, USA) was added to induce expression for 16 h at room temperature (RT), with shaking at 200 rpm. A 1 mL aliquot of culture was used for evaluating protein expression by Western blot (see below). Bacteria were recovered by spinning at 2400×*g* for 20 min and the cell pellet was used for protein extraction.

### Native and denaturing extraction methods

The *E. coli* BL21-AI culture pellet was submitted to three freezing (15 min at − 80 °C)/thawing (30 min on ice) cycles. It was then homogenized in native extraction buffer (NEB) containing 50 mM Tris, 300 mM NaCl, 25 mM imidazole, 0.1 mM EGTA and 0.25% Tween-20, at pH 8.0, and incubated for 1 h at 4 °C with constant shaking at 10 rpm using a tube rotator (Fisher Scientific, Waltham, USA). The soluble proteins (named r*Pv*DBP-RII_S_ and r*Pv*DBP-RIII/V_S_) were recovered from supernatant by spinning at 16,000×*g* for 1 h at 10 °C. Regarding the denaturing extraction method, the cell pellet was homogenized in denaturing extraction buffer (DEB) (6 M urea, 20 mM imidazole, 10 mM Tris–Cl, 100 mM NaH_2_PO_4_) to solubilize the IB; DEB was supplemented with a SIGMAFAST protease inhibitor cocktail tablet (Sigma Aldrich). The pellet was then treated with 0.1 mg/mL lysozyme overnight at 4 °C with shaking at 10 rpm. The extracted proteins (r*Pv*DBP-RII_IB_ and r*Pv*DBP-RIII/V_IB_) were recovered by spinning at 16,000×*g* for 1 h at 10 °C.

### Protein purification

The supernatants derived from total cell lysate were incubated overnight at 4 °C with Ni^2+^-NTA resin (Qiagen, Valencia, CA, USA) pre-equilibrated with NEB or DEB for purification by solid-phase affinity chromatography. The resin-protein mixture was then poured onto a chromatography column and washed with 20 mL buffer containing 0.1% Triton X-114 for eluting bacterial endotoxin, followed by 50 mL of the same buffer without detergent to detach weakly-bound proteins from the resin (contaminating molecules). Proteins were eluted with PBS containing increasing concentrations of imidazole (50, 100, 250 and 500 mM) in 3 mL fractions. Regarding proteins extracted from the IB, a dialysis step was made before elution, involving several washes with DEB containing urea in decreasing concentrations (3, 1.5, 0.75, 0.37 M and PBS). After fractions had been collected, they were analyzed by 12% SDS-PAGE and Western blot; those having a single band were dialyzed exhaustively in PBS at pH 7.2 (36 h with PBS being replaced every 4 h). The proteins were ultra-filtered, concentrated with Amicon Ultra-4 centrifugal filters (Merck Millipore, Darmstadt, Germany) and then quantified using a micro BCA protein assay kit (Thermo Scientific, Rockford, USA).

### SDS-PAGE and Western blot

The recombinant proteins contained in each fraction were treated in reducing conditions and then separated by weight on 12% polyacrylamide gel electrophoresis containing or lacking SDS, at 125 v for 2 h. The proteins were then transferred to a nitrocellulose membrane for 2 h at 10 v using Bio-Rad’s Trans-Blot SD semi-dry electrophoretic transfer cell. The membrane was washed 3 times with PBS solution containing 0.05% Tween (PBS-Tween) and then blocked for 1 h at RT with 5% (w/v) milk solution prepared in the same buffer. After 1 h incubation with peroxidase-conjugated monoclonal anti-polyhistidine antibody at a 1:4500 dilution (Sigma catalogue A7058), the reaction was revealed using a peroxidase substrate kit (Vector Laboratories, Burlingame, Canada), following the manufacturer’s recommendations. Each recombinant protein’s (r*Pv*DBP-RII and r*Pv*DBP-RIII/V) molecular mass was determined by linear regression using an XL-OptiProtein molecular weight marker (New England Biolabs, Ipswich MA, USA) as reference.

### Circular dichroism analysis

The structural features of r*Pv*DBP-RII and r*Pv*DBP-RIII/V obtained using freezing/thawing or denaturing methods were analyzed by circular dichroism (CD) in the Fundación Instituto de Inmunología de Colombia’s (FIDIC) chemistry laboratory. This required eliminating excess salts from each molecule by dialyzing 150 µg exhaustively in deionized water using a 6–8000 Dalton molecular weight cut-off (MWCO) (Spectra/Por Membrane, Spectrum Laboratories Inc.). A Jasco J-810 (JASCO Inc.) was used for CD analysis of spectra from three different batches of molecules, an average of three sweeps were taken at 20 nm/min. Spectra Manager software was used for processing the data and then analyzed using CDSSTR deconvolution software [15].

### Processing umbilical cord blood samples

Newborn umbilical cord blood (UCB) samples were collected by personnel from the Instituto de Ciencia, Biotecnología e Innovación en Salud (IDCBIS) in Bogotá, Colombia. White blood cells were then eliminated using the SEPAX system (Biosafe, Eysins, Switzerland) and their removal was confirmed by Wright staining, scanning 20 fields, using 100× objective. Duffy phenotype was typed by agglutination assay; this involved 5% erythrocytes homogenized in PBS being incubated with anti-Fy^a^ or anti-Fy^b^ antibodies (Ortho Clinical Diagnostics, Raritan, USA) for 15 min at 37 °C. After three PBS washes, cells were mixed with anti-human anti-IgG antibody and were then spun for 15 s at 1000×g. Agglutination was determined macroscopically by homogenizing each sample following the centrifugation step.

### Human reticulocyte binding assay

Flow cytometry was used for evaluating r*Pv*DBP-RII and r*Pv*DBP-RIII/V binding to human reticulocytes, in three independent assays. Briefly, 5 μL Duffy positive (Fya^−^Fyb^+^, according to agglutination assay) UCB sample was incubated with 25 μg of each recombinant protein (r*Pv*DBP-RII_S_, r*Pv*DBP-RII_IB_, r*Pv*DBP-RIII/V_S_ and r*Pv*DBP-RIII/V_IB_) for 16 h at 4 °C with shaking at 4 rpm. Following three washes with PBS containing 1% bovine serum albumin (BSA), the cells were incubated with phycoerythrin (PE) conjugated anti-histidine antibodies (MACS molecular-Miltenyi Biotec, San Diego CA, USA) in 1:40 dilution, allophycocyanin (APC-H7) conjugated mouse anti-human CD71 (Becton–Dickinson, Franklin Lakes NJ, USA) in 1:80 dilution and mouse anti-human CD45-APC (Becton–Dickinson) in 1:80 dilution for 20 min at RT in the dark. The percentage of recombinant binding to mature (normocytes) (CD71^−^CD45^−^) or immature (reticulocytes) (CD71^+^CD45^−^) erythrocytes was quantified from 1 million events acquired on a FACSCanto II cytometer (BD, San Diego CA, USA) and analyzed using FlowJo V10 software.

### HABP location

The amino acid sequences for six reticulocyte-specific HABPs were taken from a study by Ocampo et al. [16]. The r*Pv*DBP-RII 3D structure was searched in the protein data bank (PDB ID: 3RRC) [17], downloaded as a PDB file and visualized using Swiss-PdbViewer 4.1.0 software [18]. The HABPs were then sought manually and shown in different colors for locating them on the r*Pv*DBP-RII 3D model.

### Statistical analysis

Each binding assay’s mean and standard deviation were calculated from the data provided by three independent experiments. GraphPad Software (San Diego CA, USA) was used for statistical analysis (Student’s *t* test); differences were compared between each group, using a 0.05 significance level for testing the proposed hypothesis.

## Results

### *Pv*DBP has human reticulocyte binding and non-binding regions

**Fig. 1:**
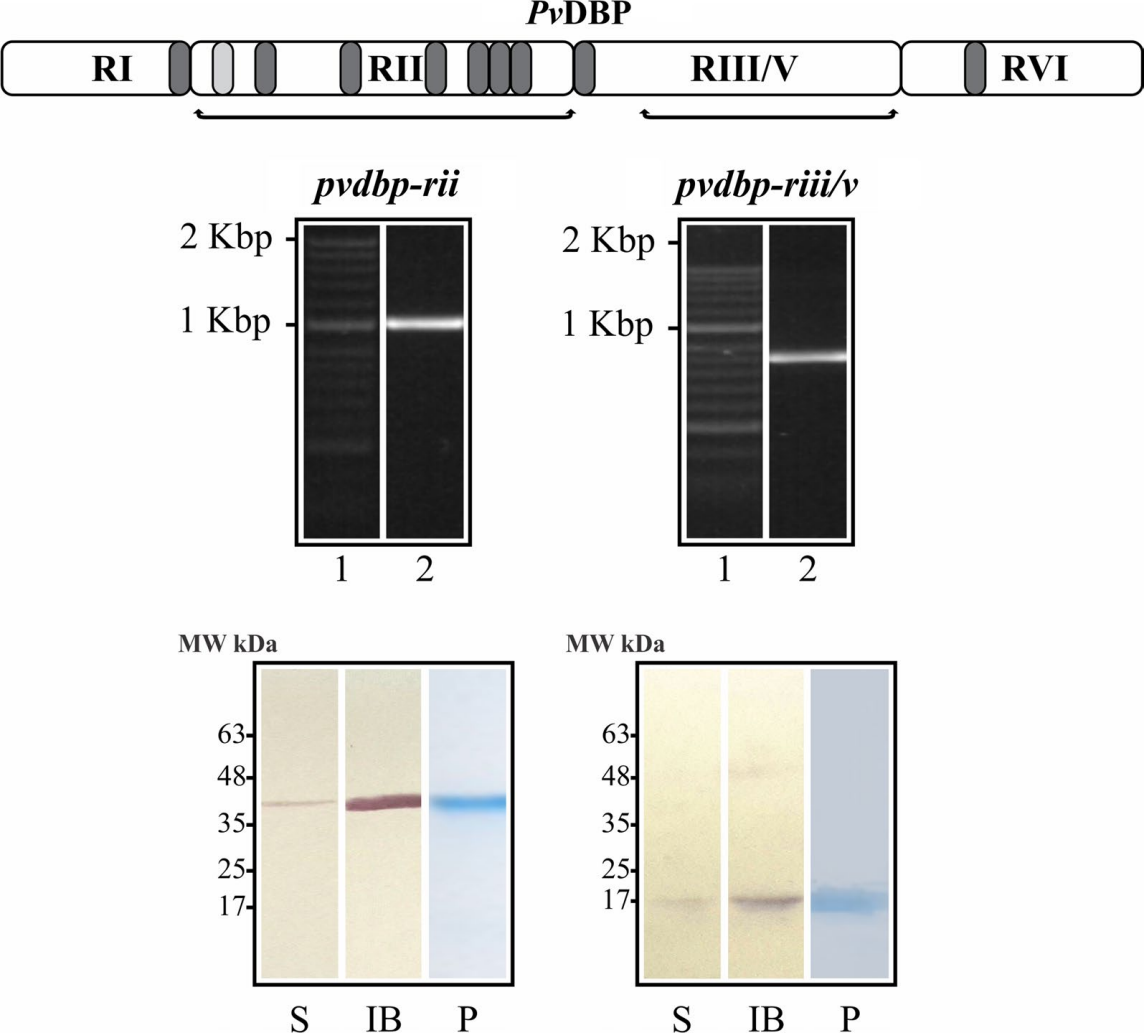
Selecting and expressing *pvdbp* gene fragments. The amplification of binding (*pvdbp*-*rii*: 553–1596 bp) and non-binding regions (*pvdbp*-*riii/v*: bp 1792–2487) encoding r*Pv*DBP-RII (aa 185–532) and r*Pv*DBP-RIII/V (aa 598–829). *Pv*DBP minimal reticulocyte binding regions are shown in dark grey; a region having normocyte and reticulocyte binding activity is shown in light grey. Agarose gel: lane 1 is the molecular weight marker. Lane 2 indicates PCR amplification of *pvdbp*-*rii* (1047 bp) and *pvdbp*-*riii/v* (699 bp) fragments. Western blot and SDS-PAGE: the molecular weight marker is shown (MW kDa), as are the molecules extracted from the soluble fraction (S) and/or inclusion bodies (IB) and purified (P)

Selecting *Pv*DBP binding controls for protein–cell interaction assays was based on that reported in the literature. Briefly, two *P. vivax dbp* gene (*pvdbp*) regions were selected for amplifying, cloned inside the pEXP5-CT vector and then transformed in TOP10 *E. coli* bacteria; these were *pvdbp*-*rii* and *pvdbp*-*riii/v*. *pvdbp*-*rii* (bp 553–1596 according the Sal-I strain sequence reported in GenBank) encoding the cysteine-rich domain (r*Pv*DBP-RII: aa 185–532) which can cause erythrocyte rosette formation when expressed on COS-7 cell surface [5]. This domain also had various high activity binding peptides (HABPs) specific for human reticulocytes (immature erythrocytes) [16], while the *pvdbp*-*riii/v* amplified region (bp 1792–2487) encoding a fragment that had no interaction whatsoever with the cells tested (r*Pv*DBP-RIII/V: aa 598–829) (Fig. 1) [5, 16].

As can be observed, a greater than 1000 bp product was obtained by PCR from the *pvdbp*-*rii* fragment and another 700 bp product from the *pvdbp*-*riii/v* fragment using gDNA from the *P. vivax* VCG-I strain, having the expected size: 1047 bp for *pvdbp*-*rii* and 699 bp for *pvdbp*-*riii/v* (Fig. 1: agarose gel electrophoresis). After obtaining the pEXP5-CT-*pvdbp*-*rii* and pEXP5-CT-*pvdbp*-*riii/v* recombinant vectors, each clone was sequenced and compared to the aa sequence of each fragment encoding r*Pv*DBP-RII and r*Pv*DBP-RIII/V among *P. vivax* Sal-I and VCG-I strains. Alignment gave three non-synonymous substitutions in c.1016A > G (p.Asp339Gly), c.1034G > A (p.Arg345His) and c.1373T > A (p.Ile458Lys) located in *Pv*DBP-RII while no mutation was observed in *Pv*DBP-RIII/V which was consistent with another report stating that region II has been seen to be variable among parasite strains from Madagascar, Ethiopia, India and Brazil [19].

### r*Pv*DBP-RII and r*Pv*DBP-RIII/V were obtained in soluble form in *E. coli*

The molecules were successfully expressed in the *E. coli* system and also extracted using freezing/thawing or denaturing method (Fig. 1: Western blot). The greatest amount of r*Pv*DBP-RII and r*Pv*DBP-RIII/V was extracted from the IB (r*Pv*DBP-RII_IB_ and r*Pv*DBP-RIII/V_IB_), this being consistent with other studies where the *Pv*DBP-RII was predominantly found in the insoluble fraction [7, 9, 10]. The molecule’s purification was determined by Coomassie blue gel staining. As can be seen, the mobility of recombinants treated with non-reducing conditions in SDS-PAGE was consistent with each one’s expected molecular weight: 42 kDa for r*Pv*DBP-RII and 16 kDa for r*Pv*DBP-RIII/V (Fig. 1: SDS-PAGE). On average, the amount of molecule extracted by the freezing/thawing method (named here r*Pv*DBP-RII_S_ and r*Pv*DBP-RIII/V_S_) was: 152 µg/L of r*Pv*DBP-RII_S_ and 479 µg/L of r*Pv*DBP-RIII/V_S_, and 267 µg/L of r*Pv*DBP-RII_IB_ and 684 µg/L of r*Pv*DBP-RIII/V_IB_ for the denaturing method.

### r*Pv*DBP-RII_S_ and r*Pv*DBP-RIII/V_S_ mainly consisted of α-helices

**Fig. 2:**
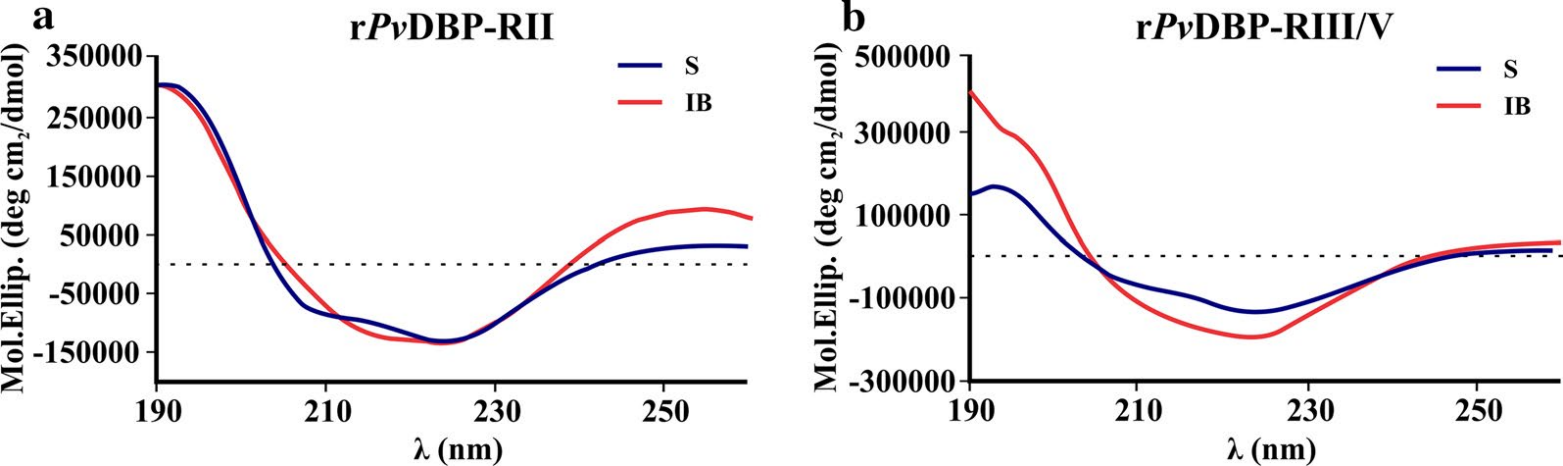
CD spectra of two *Pv*DBP-derived recombinant proteins. The CD spectra for r*Pv*DBP-RII (**a**) and r*Pv*DBP-RIII/V (**b**) obtained from soluble (S) (blue line) or inclusion body (IB) (red line) fractions

Each molecule’s secondary structure and folding properties were evaluated by CD spectroscopy. The spectra for r*Pv*DBP-RII_S_ and r*Pv*DBP-RIII/V_S_ (but not for r*Pv*DBP-RII_IB_ and r*Pv*DBP-RIII/V_IB_) showed a characteristic α-helical conformation pattern, characterized by having a 192 nm maxima and 208 and 222 nm minima (Fig. 2). CDSSTR deconvolution software analysis for determining structural elements showed that r*Pv*DBP-RII_S_ consisted of 76.8% α-helices, 11.1% β-sheets and 5.9% β-turns while r*Pv*DBP-RIII/V_S_ had 67.3% α-helices, 11.5% β-sheets and 7.4% β-turns.

### r*Pv*DBP-RII_S_ and r*Pv*DBP-RIII/V_S_ binding to reticulocytes and normocytes

**Fig. 3:**
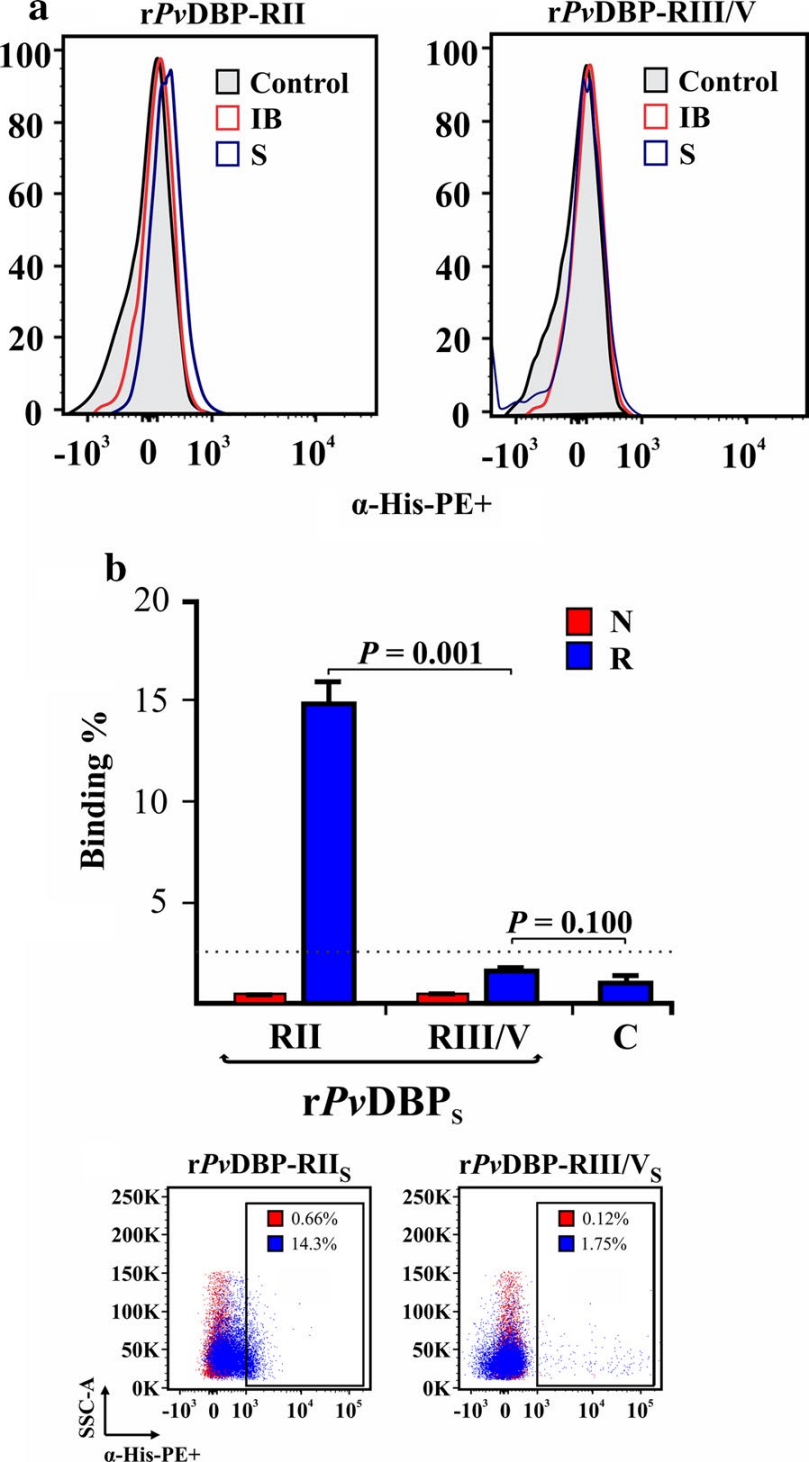
r*Pv*DBP-RII and r*Pv*DBP-RIII/V erythrocyte binding activity. **a** A representative histogram from two independent experiments showing the PE signal (α-His-PE^+^) for reticulocyte binding assay using r*Pv*DBP-RII and r*Pv*DBP-RIII/V extracted from soluble (S) or inclusion body (IB) fractions compared to control (CD71^+^CD45^–^PE^–^). **b** r*Pv*DBP-RII_S_ and r*Pv*DBP-RIII/V_S_ human normocyte (N) and reticulocyte (R) binding percentages. A characteristic dot plot used for building the bar chart is shown at the bottom of the figure

**Fig. 4:**
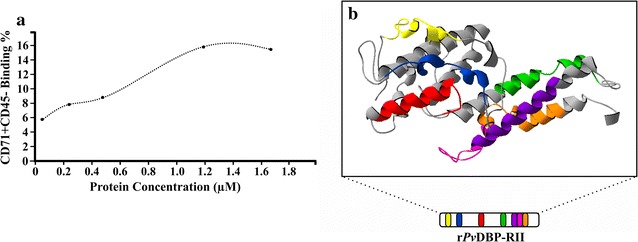
r*Pv*DBP-RII saturation (**a**) and binding site location (**b**) analysis. **a** The protein concentration (x axis) and reticulocyte (CD71^+^CD45^−^) binding percentage. **b** HABPs location on r*Pv*DBP-RII 3D structure is shown in different colors

Protein–cell interaction was evaluated by flow cytometry, using r*Pv*DBP-RII and r*Pv*DBP-RIII/V obtained from each extraction method (freezing/thawing or denaturing) and white cell-depleted newborn umbilical cord blood. There was a displacement in the histogram when comparing r*Pv*DBP-RII_S_ binding to human reticulocytes (CD71^+^CD45^−^) unlike r*Pv*DBP-RII_IB_ (Fig. 3a) (*t* test: *t*_(4)_ = 30.50, *P* = 0.001). Regarding r*Pv*DBP-RIII/V_S_ and r*Pv*DBP-RIII/V_IB_, there was no statistical difference concerning binding compared to negative control (r*Pv*DBP-RIII/V_S_: *t* test: *t*_(4)_ = 2.13, *P* = 0.100; r*Pv*DBP-RIII/V_IB_: *t* test: *t*_(4)_ = 1.79, *P* = 0.147). Particularly interesting was the fact that r*Pv*DBP-RII_S_ bound more to reticulocytes (CD71^+^CD45^−^) (15.1% ± 1.06) compared to normocytes (CD71^−^CD45^−^) (0.72% ± 0.08) (Fig. 3b). Furthermore, r*Pv*DBP-RII_S_ binding to human reticulocytes was greater than that observed for r*Pv*DBP-RIII/V_S_ (*t* test: *t*_(4)_ = 30.80, *P* = 0.001) and increased in a concentration-dependent manner, becoming saturated at 1.2 µM (Fig. 4a).

### r*Pv*DBP-RII_S_ reticulocyte binding correlates with its α-helical content

r*Pv*DBP-RII 3D structure was analyzed for localizing each HABP to reticulocytes reported in the literature [16], using Swiss-PdbViewer software [18]. Analysis of r*Pv*DBP-RII minimal binding regions overlapping in the 3D structure of the molecule reported in the PBD database [17], revealed that most had an α-helical structure (Fig. 4b), suggesting that r*Pv*DBP-RII_S_ reticulocyte binding activity is correlated with its structural features (mainly α-helical content).

## Discussion

An in-depth understanding of parasite-host interactions is important to comprehend the complex machinery involved in some microorganisms’ invasion of their target cells and thus establish appropriate control methods. Concerning *P. vivax*, parasite protein interactions with their target cells (reticulocytes) has been studied with molecules obtained from eukaryote (i.e. *Sf* insect cells [7, 20], COS-7 cells [21], or wheat germ cell-free system [22]) or prokaryote (mainly *E. coli*) [23, 24] systems; the latter has been most used due to its methodological and economic advantages. In spite of the forgoing, the greatest challenge in using the *E. coli* system lies in the difficulty in obtaining functional molecules. An easy-to-use, fast and economic technique for producing and extracting two controls which would be useful regarding protein–cell interaction techniques was thus standardized here; it was based on screening a molecule which is important for *P. vivax* binding to human reticulocytes.

This strategy consisted of selecting DBP for expressing and obtaining binding (r*Pv*DBP-RII) and non-binding (r*Pv*DBP-RIII/V) fragments in the *E. coli* system [5, 16] (Fig. 1). The pEXP5-CT/TOPO vector was thus used since it contains a high-level T7 inducible promoter, a pUC origin for plasmid high-copy replication and maintenance in *E. coli*, an efficient cloning site and a C-terminal fusion tag for detecting and purifying recombinant fusion proteins. In spite of the problem regarding insolubility which has been reported when the r*Pv*DBP-RII is expressed in *E. coli* [7, 9], it was obtained, as well as the III/V region, using the freezing/thawing process (Fig. 1). This was probably due to the incubation conditions used here (25 °C for 16 h), since it has been reported that a smaller amount of IB is produced as the temperature becomes reduced, thereby favoring soluble protein production [25, 26]. Even though a greater amount of r*Pv*DBP-RII_IB_ and r*Pv*DBP-RIII/V_IB_ was obtained, these molecules did not have a characteristic α-helix structure pattern compared to r*Pv*DBP-RII_S_ and r*Pv*DBP-RIII/V_S_ (Fig. 2), supporting the notion that extensive dialysis with PBS is not enough for recovering an appropriate structure for such molecules extracted from IB. This result highlighted the fact that the refolding method is essential for obtaining r*Pv*DBP-RII (extracted from IB) in fully functional form, as has been described by Singh et al. [9]. Thus, even though the advantage of expression in IB lies in producing a greater amount of the molecule, correct protein folding is difficult to recover by a common dialysis method.

The molecule’s human erythrocyte binding activity was determined and quantified by flow cytometry to investigate whether structure played an important role in such function. Duffy+ (FYa^−^FYb^+^) human blood (*P. vivax* preferred population [27]) was used for this and antibodies directed against each recombinant protein (anti-His-PE) or against the reticulocyte population (anti-CD71-APC-H7) and white blood cells (anti-CD45-APC). The transferrin receptor (CD71) was used as reticulocyte marker, as previously reported [27], while CD45 receptor labelling was included in the assay so as to exclude activated lymphocytes from the analysis (they express CD71 on their surface also, but contrary to reticulocytes, they are CD45^+^). The molecules extracted by freezing/thawing process were capable of binding target cells, unlike those extracted using the denaturing method (Fig. 3). A greater percentage of r*Pv*DBP-RII_S_ bound to human reticulocytes rather than normocytes (Fig. 3a), which can be substantiated by the fact that it consists of six reticulocyte HABPs, as demonstrated by Ocampo et al. [16]. Regarding r*Pv*DBP-RIII/V_S_, its binding was similar to that demonstrated for control (CD71^+^CD45^–^PE^–^ cells), coinciding with previous studies showing that the region expressed on COS-7 cell surface did not cause rosette formation or have reticulocyte or normocyte HABPs [5, 16]. The fact that only r*Pv*DBP-RII_S_ (77% α-helix content) bound to reticulocytes but not r*Pv*DBP-RII_IB_ (which did not show a characteristic α-helix structure) highlighted that the secondary structure was important for the molecule’s binding activity.

The 3D structure reported by Batchelor et al. [28] was thus analyzed for locating structural elements and determining whether they could have any importance regarding binding by comparing them with previously identified r*Pv*DBP-RII minimal binding regions [16] (Fig. 1). Interestingly, it was observed that most r*Pv*DBP-RII HABPs reported have α-helix structures (Fig. 4b) which, added to the CD analysis and protein–cell interaction here reported (Figs. 2, 3), suggest that such structures are extremely important for r*Pv*DBP-RII_S_ human reticulocyte binding activity; these structures could be affected by treatment with urea, as reported in this and previous studies [7, 8].

The forgoing supports the idea that the methodology described here could be used for obtaining parasite molecules in soluble and functional form, as described recently [29–31] avoiding the denaturation and refolding steps commonly used. However, it does not mean that this should become a rule for all *P. vivax* proteins, because it has also been shown that some of them can be obtained functionally from IB, such as some molecules belonging to the *Pv*-fam-a [23], *Pv*TRAg [24] and *Pv*RON families [31]. The results highlight r*Pv*DBP-RII_S_ and r*Pv*DBP-RIII/V_S_ as optimal controls to be used in *P. vivax* protein-target cell interaction assays. Given that no negative control such as r*Pv*DBP-RIII/V has been studied in detail to date by rosetting assay [5], screening minimal binding regions [16] and/or flow cytometry (shown here), this could be useful to establish a baseline regarding the positive interaction between parasite proteins and their target cells.

## Conclusions

The methodology described may provide a starting point for *P. vivax* protein production and extraction in soluble form, with a proper folding in the *E. coli* system; it could be useful in biotechnological applications. This is the first report showing how the r*Pv*DBP-RII domain can be obtained in soluble form avoiding denaturation and refolding steps and also highlighting the usefulness of the r*Pv*DBP-RIII/V fragment as a non-binding control in protein–cell interaction assays. This methodology could be used for determining the capability of various *P. vivax* molecules to bind to human reticulocytes and thus screen their proteins or regions which could be used as components of a vaccine against the species.

## Abbreviations

*Pv*DBP: *Plasmodium vivax* Duffy binding protein
DARC: Duffy antigen receptor for chemokines
RII: region II
Cys: cysteine
r: recombinant
HABP: high activity binding peptide
CD: circular dichroism
S: soluble
IB: inclusion bodies
Sal-I: Salvador I
*pvdbp*: *Plasmodium vivax dbp*
LB: Luria–Bertani
VCG-I: Vivax Colombia Guaviare 1
RT: room temperature
NEB: native extraction buffer
DEB: denaturing extraction buffer
FIDIC: Fundación Instituto de Inmunología de Colombia
Duffy+: Duffy positive
